# Monkey visual attention does not fall into the uncanny valley

**DOI:** 10.1038/s41598-022-14615-x

**Published:** 2022-07-11

**Authors:** Sarah B. Carp, Anthony C. Santistevan, Christopher J. Machado, Alexander M. Whitaker, Brittany L. Aguilar, Eliza Bliss-Moreau

**Affiliations:** 1 California National Primate Research Center, University of California Davis, Davis, CA 95616 USA; 2 Department of Psychology, University of California Davis, Davis, CA 95616 USA; 3 Department of Psychiatry and Behavioral Sciences, University of California Davis, Davis, CA 95616 USA; 4 Present Address: Cuesta College, San Luis Obispo, USA; 5 Present Address: New York Academy of Sciences, New York, USA

**Keywords:** Psychology, Social neuroscience, Attention

## Abstract

Very humanlike artificial agents can induce feelings of uneasiness in human perceivers. Stimuli that generate this response are said to occupy “the uncanny valley”. Given inconsistent findings in the literature, whether or not nonhuman animals experience the uncanny valley is unclear. Here, we recorded the visual attention of eleven male rhesus monkeys as they viewed faces varying in realness across five levels, with visual attention measured by both number and duration of visual fixations on faces as a whole and on areas of interest within the faces (e.g., eyes, mouth). Face stimuli varied in terms of the realism of the image and behavior depicted by the face (lipsmack, threat, bared teeth, and neutral). We largely found no support that rhesus monkeys perceive an uncanny valley when viewing our stimuli; however, monkeys did generally pay more attention to eyes and less attention to mouths in real images compared to less realistic images. Across all stimuli, monkeys’ visual attention was drawn to the mouths of images when teeth were visible. These findings suggest that rhesus monkeys in our study did not display an uncanny valley effect when viewing realistic stimuli but did percieve affective information depicted by faces regardless of how real those faces appear.

## Introduction

Machine or artificial agents appear more attractive the more humanlike they become, until they are so human-looking that they instead elicit feelings of creepiness or uncanniness in people. This nonlinear dip in affinity, known as the uncanny valley (UV), first hypothesized by Mori (1970)^1^, develops during childhood^2–4^ and has been well-documented in adults (e.g.,^5–9^). While the existence of the UV is well-documented in published scientific literature, our understanding of the factors that contribute to a UV as well as its evolutionary origins remain elusive.

Why would it be that faces that look *too* realistic are perceived as creepy or weird? One group of evolutionary hypotheses suggests that the UV arose from selection pressures such as pathogen or danger avoidance, wherein humans evolved a repulsion to the appearance of illness or disease. According to these hypotheses, human faces that appear close, but “not-quite-right”, activate an avoidance response^10–12^. Additionally, error detection in visual processing may occur when small differences between an artificial and real human face are perceived as defects from normal which are interpreted as unattractive or creepy in order to encourage avoidance of individuals which ultimately has fitness consequences^13,14^.

Alternatively, there are several hypotheses that suggest that the capacity for self-referential thought and having an understanding about what it means to be human are required to perceive the UV. In this view, the UV results from violations of our acquired understanding of what makes humans unique from machines^13,14^. This hypothesis is consistent with evidence that the greater a robot’s perceived capacity for feeling, the more unnerving they are to humans^15^. These ideas form the basis of competing hypotheses that allow for the evaluation of potential evolutionary arguments about how the UV as a perceptual bias resulted from selection pressures. If the UV serves as a pathogen avoidance detection mechanism, then we might expect to see it in other non-human species—especially those with low neophobia that live in broad environmental niches who are likely to make greater contact with unknown pathogens and rely heavily on faces for understanding identity and as social communication tools (e.g., such as monkeys from the genus *Macaca*, long-tailed macaques^16^; bonnet macaques^17^; rhesus macaques^18,19^; reviewed in^20^; for a review of monkey facial behavior see^21^). Furthermore, rhesus monkeys and humans use similar attentional strategies when processing facial stimuli^22^, suggesting that underlying biological processes used to evaluate facial stimuli may also be conserved. If, on the other hand, the UV is the result of a violation of knowledge about what it means to be human and our ability to mentally separate ourselves from machines, then the UV is likely to be a uniquely human phenomenon without deep evolutionary roots. While some non-human animals do have the capacity for self-recognition (or can be trained to recognize themselves), it is likely that complex cognitive comparisons that question the nature of self-hood by comparing the self to machines are outside the scope of most other species’ ability^23–26^.

A few existing reports speak to whether macaque monkeys experience the UV but provide conflicting results. For example, data from one previous study with long-tailed macaques suggests that macaques also experience the UV^12^. In that study, five adult, male, long-tailed macaques were shown images of monkey heads that varied in “realness”. The observer monkeys generated the greatest number of visual fixations (measured via eye tracker) on both real photos of other monkeys and the least realistic artificial images, and generated the least number of fixations on the screen displaying a picture of a monkey head that was proposed by the authors to be in the UV. The stimuli used in this experiment for realistic and unrealistic stimuli were very different and the extent to which the unrealistic image looked like a monkey was questionable. The unrealistic image was grayscale with red pupils and no hair, and the highly realistic image was in color with realistic colored eyes and smoother visual features, also with no hair. The extreme visual differences between the unrealistic and realistic artificial faces have been critiqued as the foundation for a potential alternative explanation for their results, as the two images may have elicited differential interest or curiosity as a result of novelty rather than eeriness^27^.

Two more recent reports used more realistic stimuli^27,28^, although they differed in terms of some specific features. In one study, when adult long-tailed macaques and rhesus macaques viewed the study stimuli images there was no evidence for a UV, with no difference in looking time between the unrealistic artificial, realistic artificial, and real images. Unlike the report by^12^, these faces all depicted a neutral facial behavior with averted gaze. However, the stimulus images were generated from the skull of a long-tailed macaque even though they were tested with rhesus macaque subjects. A second research group studying rhesus macaques used a wider range of five levels of realism expanding from highly unrealistic (virtual wire framing) to real images all generated from a rhesus macaque model and found a dip in attention (a UV) to more unrealistic faces^28^. Like the stimuli used in^12^ and in^27^ they did not include all features across all levels of the stimuli (e.g., only the most realistic face included hair and realistic coloring was not maintained across all levels of manipulation), leaving open questions about whether the UV is a perceptual bias evolved across species that is dependent on certain features or the particular type of information that is available in images.

The present report is a conceptual replication of the previous UV studies in monkeys, particularly inspired by the findings of Steckenfinger and Ghazanfar^12^ [Note: Data for this study were collected prior to the publication of^27^ and^28^ and so we have integrated discussion of those papers into this report, but our original hypotheses were inspired by^12^.] By conceptual replication, we mean that key features of the original methods were varied but the general approach and the question tested remained the same insofar as we tested adult monkeys on real and computer-generated artificial faces that varied by facial behavior, although we emphasized making the face stimuli more realistic than those previously used. We maintained hair across all levels of realism and also used realistic coloring throughout all levels of realism in our stimuli set, unlike some previous assessments of the uncanny valley^12,27,28^. As a result of these choices that we made when generating the stimuli, all levels of realism had the same types of information (e.g., information about hair, eyes, teeth (when applicable) and coloration) rather than manipulating levels of realism by manipulation what type of information was available as in Refs.^12,27,28^.

In the present study, we evaluated whether eleven rhesus monkeys (*Macaca mulatta*) (as in Refs.^27,28^), rather than long-tailed, (*Macaca fasciularis*) (as in Refs.^12,27^), showed evidence of a UV in their visual behavior toward facial stimuli. As in Refs.^12,28^, we used fixation frequency and fixation duration as our measures of visual attention. We replicated the statistical analyses that^12^ carried out but also expanded the analyses to include measures related to how facial behavior depicted in the stimuli and degree of face realness may alter looking patterns in specific areas of interest on the face, such as the eyes and mouth similar to analyses in Ref.^28^. Aspects of our study and analysis plan were pre-registered (https://osf.io/bj6ek).

## Results

**Figure 1:**
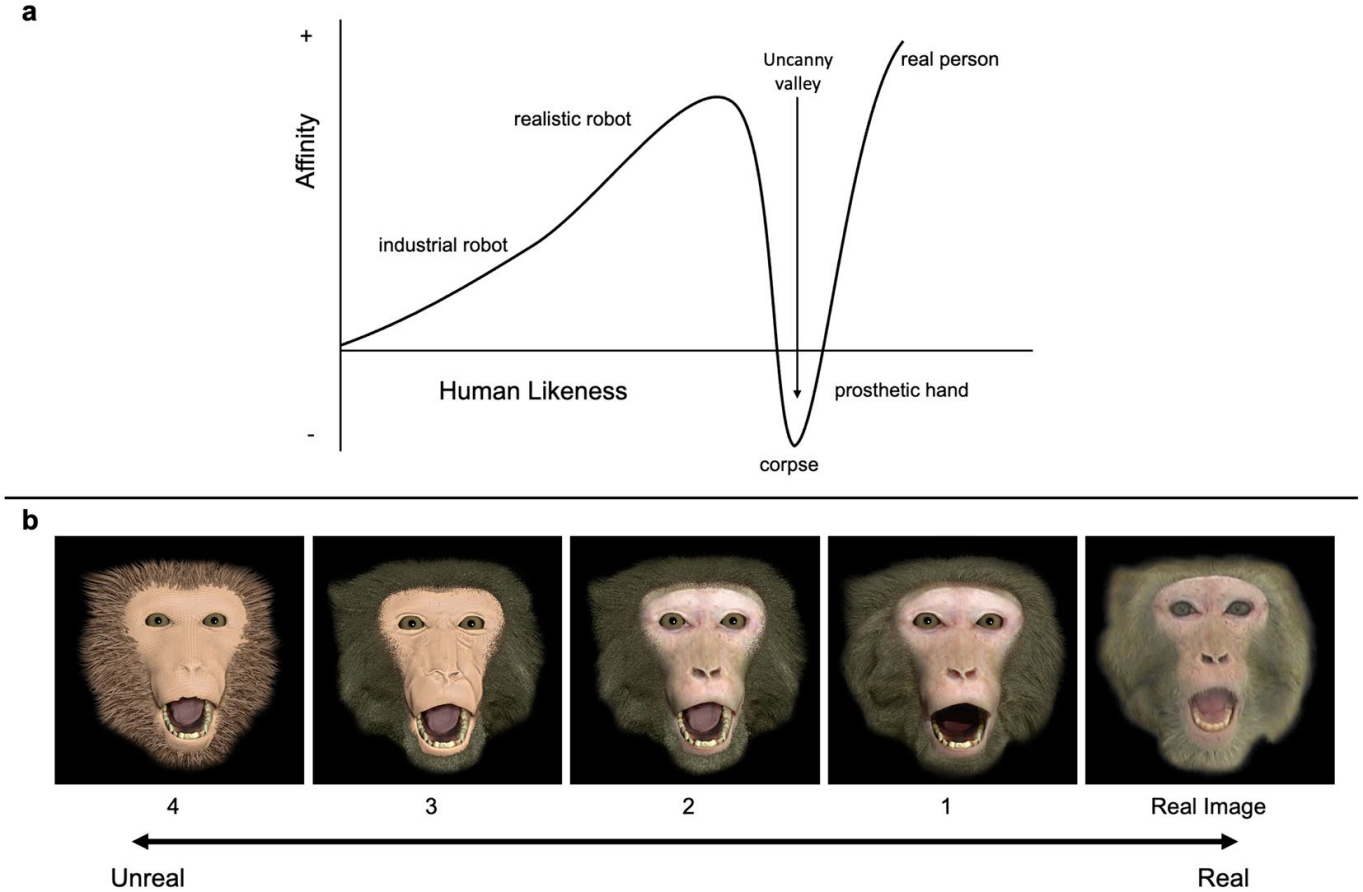
Visual descriptions of the UV hypothesis and stimuli used in the present experiment. (**a**) The UV hypothesis developed by Mori (1970) suggests that as human likeness increases so does affinity, until a certain threshold is reached at which point affinity quickly drops (producing the UV) before spiking up again to its highest level for a real human image. Figure modified from^1,12^. (**b**) Examples of stimulus images we used for a threat face ranging from a Real image of a monkey through increased levels of manipulation (Faces 1 through 4, where Face 4 is the least realistic) to create a realness continuum for each stimulus.

To evaluate whether rhesus monkeys experience a UV when perceiving faces, we created an artificial face set which varied systematically in terms of how realistic they looked. In order to determine the sensitivity of rhesus monkeys to the UV, we used five different realism levels defined via the process of rendering the images in the software program that produced them, detailed in supplementary information (Supplementary Methods S1, Fig. S1) and in Fig. 1. Eleven adult male rhesus monkeys watched the face set while their gaze was tracked using an infrared eye tracker. We then carried out a series of analyses as detailed in our pre-registration^29^. As in Steckenfinger & Ghazanfar^12^, we began by evaluating overall time that the monkeys looked at the face stimuli, with looking quantified with two variables: fixation frequency and fixation duration.

### Conceptual replication

Steckenfinger and Ghazanfar^12^ computed the mean-normalized fixation frequency and/or fixation duration directed toward each stimulus (a floating monkey face with a black/white background), pooled across facial behaviors (e.g., coo, scream, neutral) and evaluated the overall effect as well as pairwise effects for each analysis that reached significance. We replicated this strategy and pooled across monkey identity given that we used four different monkey identities. We first analyzed the looking behavior directed at the entire screen in order to best match the analysis done by Ref.^12^. While the original report^12^ found a UV effect for both fixation count and duration, we found no UV-related effect in the number of fixations (Freidman Test, Friedman Statistic, *Q*(4, *n* = 11) = 1.88, *p* = 0.758), or duration of fixations (Freidman Test, Friedman Statistic, *Q*(4, *n* = 11) = 8.51, *p* = 0.075), suggesting that there was no evidence that our monkeys perceived a UV.

Our area of interest (AOI) protocol allowed us to extract additional information, including when monkeys were looking at the computer screen but not directly at the faces (i.e., fixation frequencies and durations that were not on the face) and when monkeys were looking at specific regions of the face. All data were processed as described above; Dunn’s posttest was used to correct for multiple comparisons. First, we assessed the time monkeys spent looking at the face, but not at the rest of the screen. There was not a significant effect of the realness of the stimuli on the average frequency of fixations on the stimulus face (Friedman Test, Friedman Statistic, *Q*(4, *n* = 11) = 4.44, *p* = 0.350) or on fixation durations (Friedman Test, Friedman Statistic, *Q*(4, *n* = 11) = 4.00, *p* = 0.406). In the original report^12^ there was no distinction between the face and the rest of the screen; looking time at the screen was analyzed as time spent looking at any region within the screen. Because monkeys were able to look completely away from the computer screen, time looking at the non-face region is not a perfect inverse to time looking at the face. Looking time at the screen and not the face provides information about when the animal is attending to the task but avoiding looking at the face. There was a significant effect of face realness on fixation frequency toward the region outside of the face (Friedman Test, Friedman Statistic, *Q*(4, *n* = 11) = 11.85, *p* = 0.019) indicating variability across levels. However, there were no significant differences between any individual realness conditions as per the post hoc tests and the overall pattern of means was not suggestive of the UV (all *p* > 0.05). There was not an effect of face realness on fixation duration on the region outside the face (Friedman Test, Friedman Statistic, *Q*(4, n = 11) = 7.2, *p* = 0.126).

### Further evaluation for the existence of an uncanny valley

We carried out additional analyses to examine looking behavior at each feature of the face (eyes, mouth) because existing data demonstrate that monkeys scan individual features of faces differently (e.g., variation between looking at eyes versus mouth)^30–32^ and some of the computer generated face features may look particularly artificial (e.g., teeth) to the monkeys. We also assessed if looking preferences were driven by specific affect-related facial behaviors (e.g., threat, bared teeth, neutral, lipsmack). These analyses are similar to those carried out by Ref.^28^. The Friedman test on *average* number of fixations employed in our initial analyses ignores trial-level variability, leading to statistically inefficient estimation of effects of face realness/affective properties on looking patterns (that is, aggregating reduces power to detect differences) (c.f. Ref.^33^).

For each AOI (eyes, mouth, outside) we conducted further analyses examining the effect of facial behavior and face realness on looking behavior. Using generalized linear mixed effects models in R (version 4.0.2) we analyzed two aspects of behavior for each AOI. In the first model we examined the frequency of fixations directed toward the AOI. Next, we analyzed fixation duration using a model in which we analyzed fixation durations only for trials in which the subject fixated on the AOI. All models included degree of face realness, facial behavior and their interaction, as well as test day to assess habituation over the five days of testing. We also included subject identity as a random factor. All data were analyzed at the trial level for each animal. All models are summarized in tables in the Supplementary Information (Supplementary Tables S1–S12) and the pattern of results relevant to the UV and effect of facial behavior are summarized in Table 1. Only results relevant to the UV are reported in the text.

**Table 1:** Summary of results relevant to the UV.

	*Face*	*Outside*	*Entire Screen*	**Eyes**^**1**^		Mouth	**Outside**
**(a) Fixation frequency**							
Face realness	NS	NS	NS	**Facial behavior**	**Face realness**	Real < 2,4	**Real, 3, 4 < 1** 4 < 2
				Neutral	4 < 2, Real **1 < Real, 2**		
				Lipsmack	4 < 1, Real		
				Bared Teeth	3 < Real		
				Threat	3 < Real 4 < Real, 1		
Facial behavior	–	–	–	See above interaction		Neutral < Threat, Bared Teeth Lipsmack, Threat, Neutral < Bared Teeth	Bared Teeth < Neutral
Evidence for UV?	No	No	No	Minimal from bolded result		No	Minimal from bolded result

	*Face*	*Outside*	*Entire Screen*	**Eyes**	**Mouth**	**Outside**	
**(b) Fixation duration**							
Face realness	NS	NS	NS	3, 4 < Real 4 < 1	Real < 1, 2, 3, 4	NS	
Facial behavior	–	–	–	Bared Teeth < Neutral	Neutral < Threat, Bared Teeth	Bared Teeth < Neutral	
Evidence for UV?	No	No	No	No	No	No	

Outcome variables are fixation frequency (**a**) and fixation duration (**b**). Headings in italics represent replication analyses conducted with Friedman’s tests while headings in bold represent results from the expanded AOI analysis. The “–" denotes that the factor was not assessed in the model. Overall, there was very minimal support for the hypothesis that rhesus monkeys are sensitive to the UV effect (bolded results). ^1^Results from a significant interaction between facial behavior and face realness are summarized within the cell for Face Realness.

**Figure 2:**
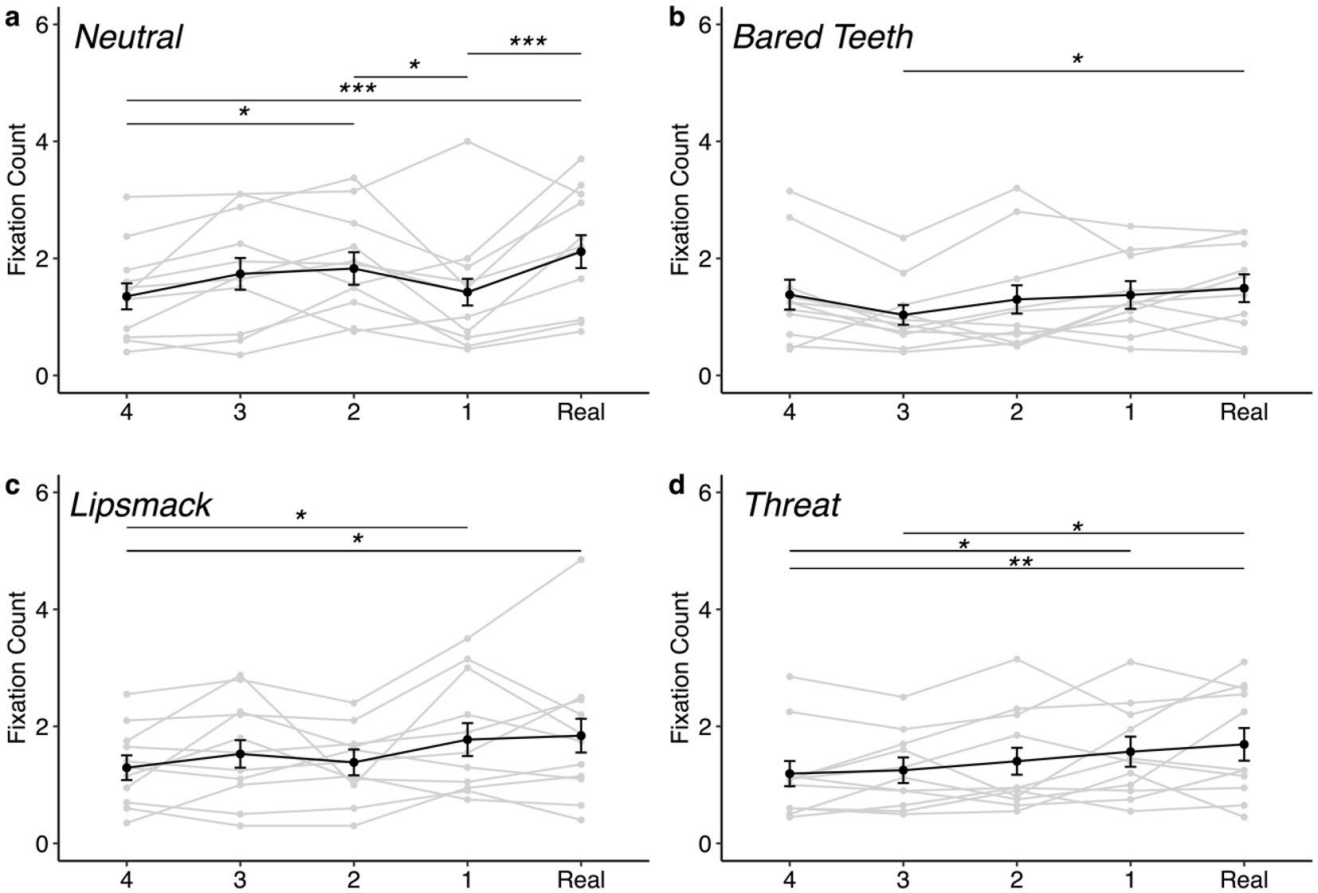
Frequency of fixations on the eyes of the face stimulus as a function of stimulus realness separated by facial behavior of the stimulus. Each subject’s mean behavior is depicted by a gray line and the black line represents the average across all subjects. Each panel represents data from 1040 individual trials. (**a**) Visual attention to the eyes of faces displaying neutral (or no) behavior. Minimal evidence for a UV effect in which subjects fixate less frequently on the Face 1 stimulus than either the Real (*z* = − 4.102, *p* =  < 0.001) or Face 2 (*z* = 2.75, *p* = 0.047) stimulus. (**b**) Visual attention to the eyes of faces displaying a bared teeth display revealed no evidence for a UV effect. Subjects looked at the eyes of the real image more frequently compared to the less realistic Face 3 (*z* = − 3.15, *p* = 0.014), but there were no other significant differences between stimulus realness. (**c**) Visual attention to the eyes of faces displaying a lipsmack display reveals no evidence for a UV effect. Subjects looked at the eyes less frequently in the least real image Face 4 compared to either the Real image (*z* = − 3.21, *p* = 0.012) or most real, but still manipulated image, Face 1 (*z* = − 2.92, *p* = 0.029), indicating reduced attention to less realistic eyes. (**d**) Visual attention to the eyes of faces displaying a threat display reveals no evidence for a UV effect. Subjects looked at the eyes fewer times in the least real image Face 4 compared to either the Real image (*z* = − 3.35, *p* = 0.007) or most realistic of the manipulated images, Face 1 (*z* = − 2.89, *p* = 0.032). Subjects also looked at the eyes fewer times in the Face 3 image compared to the Real image (*z* = − 2.86, *p* = 0.034). *p < 0.05, **p < 0.01, ***p < 0.001. Error bars represent mean ± 95% Confidence Interval.

**Figure 3:**
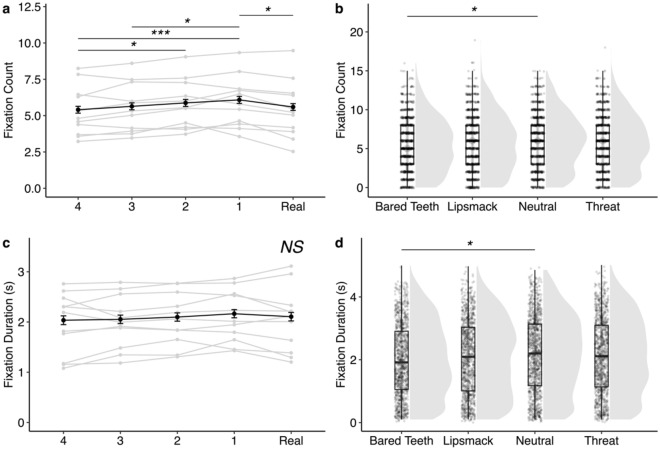
Visual attention directed to the screen but not the face. Fixation count data are in top panels and fixation duration data in bottom panels. In plots on the left (**a**,**c**), gray lines represent average behavior of a given subject pooled across facial behaviors and the black line represents average behavior across all subjects. In plots on the right (**b**,**d**), individual data points are overlaid on boxplots with violin plot density functions on the right for each facial behavior pooled across face realness levels. (**a**) Subjects looked outside more times when the stimulus image was the most realistic of the manipulated images Face 1 compared to when it was the Real image (*z* = − 3.23, *p* = 0.011), Face 3 (*z* = 2.80, *p* = 0.041) or the least realistic Face 4 (*z* = 4.34, *p* < 0.001), suggesting that there may be some degree of aversion to the highly realistic Face 1 image. Monkeys also fixated outside the stimulus more times when the image was Face 2 than when it was the least realistic Face 4 (*z* = 3.06, *p* = 0.019). Data are summarized across 832 individual trials per face realness level. (**b**) Subjects looked outside the face stimulus more times when the facial behavior of the stimulus was neutral compared to when it was bared teeth (*z* = − 2.91, *p* = 0.019). Data represent 1040 trials per facial behavior. (**c**) Subjects did not vary the duration of their fixations on the outside of the stimulus based on stimulus realness. Data are averaged across 771–794 individual trials per level of face realness. (**d**) Subjects fixated on the outside for longer when the stimulus displayed a neutral facial behavior compared to when it displayed bared teeth (*z* = 2.70, *p* = 0.035). Data represent 964–988 trials per facial behavior. *p < 0.05, **p < 0.01, ***p < 0.001. Error bars represent mean ± 95% Confidence Interval.

Briefly, there was only minimal evidence to support rhesus monkeys being sensitive to the UV effect. When subjects looked at the eyes of stimuli depicting a neutral facial behavior, subjects fixated on the eyes fewer times in the least realistic image (Face 4) compared to the more realistic versions (Face 2, *z* = -3.062, *p* = 0.019; or the Real face stimuli, *z* = -4.41, *p* < 0.001). Subjects also fixated on the eyes fewer times in the most real, but still manipulated image (Face 1) compared to either the Real image (*z* = -4.102, *p* < 0.001) or the image that was one level less realistic (Face 2, *z* = 2.75, *p* = 0.047, see Fig. 2a). This decrease in number of fixations between the Real and Face 1 and between Face 1 and Face 2 does lend support to the hypothesis of a UV effect in behavior directed toward the eye AOI for neutral facial behaviors. This pattern was not observed for other facial behaviors (see Fig. 2b–d). Additionally, subjects looked outside more times, regardless of affective display of the stimulus face, when the stimulus image was the most realistic of the manipulated images (Face 1) compared to when it was the Real image (z = − 3.23, *p* = 0.011) or when it was Face 3 (*z* = 2.80, *p* = 0.041) or the least realistic Face 4 (*z* = 4.34, *p* < 0.001; Fig. 3a), suggesting that there may be some degree of aversion to the highly realistic (Face 1) image which would be supportive of a UV.

**Figure 4:**
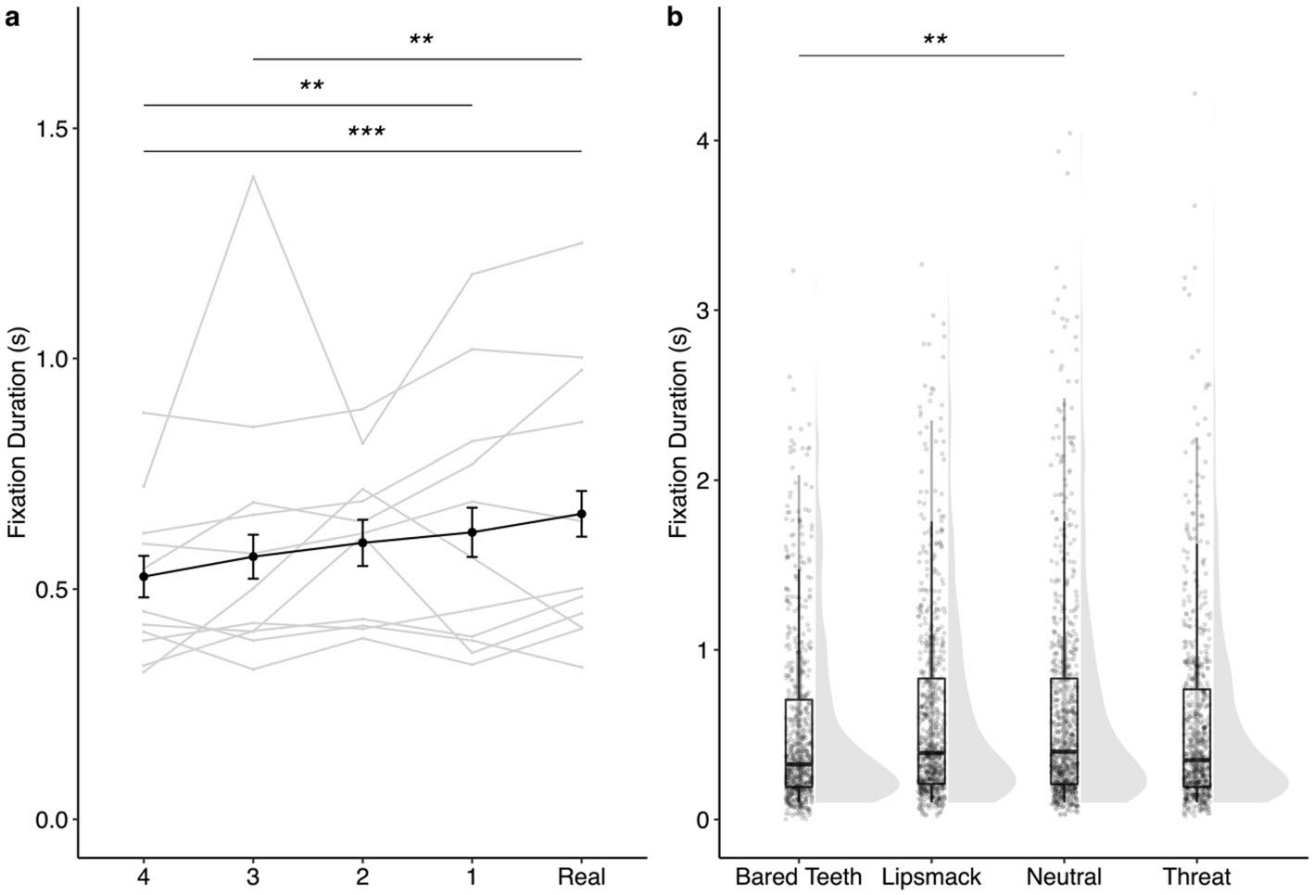
Duration of fixations directed at the eyes of the face stimuli. (**a**) Gray lines represent average fixation duration of a given subject across all facial behaviors. The black line represents average fixation duration across all subjects. Data in this panel represent 514–573 individual trials per Face Realness level. Subjects looked longer at the eyes in the Real image compared to either the least realistic Face 4 (*z* = 4.31, *p* < 0.001) or Face 3 (*z* = 3.51, *p* = 0.004). Subjects also looked longer at the eyes in the most realistic manipulated Face 1 image compared to the least realistic Face 4 image (*z* = 3.41, *p* = 0.005), which contradicts what would be expected for a UV in which Face 1 is anticipated to receive the least amount of attention. (**b**) Plots represent individual data points (622–722 trials per facial behavior) overlaid on boxplots on the left with violin plot density functions on the right for each facial behavior. Monkeys altered how long they looked at the eyes based on facial behavior of the stimulus, such that monkeys spent less time looking at the eyes of a face with the bared teeth display than one displaying a neutral facial behavior (*z* = 2.40, *p* = 0.004). *p < 0.05, **p < 0.01, ***p < 0.001. Error bars represent mean ± 95% confidence Interval.

**Figure 5:**
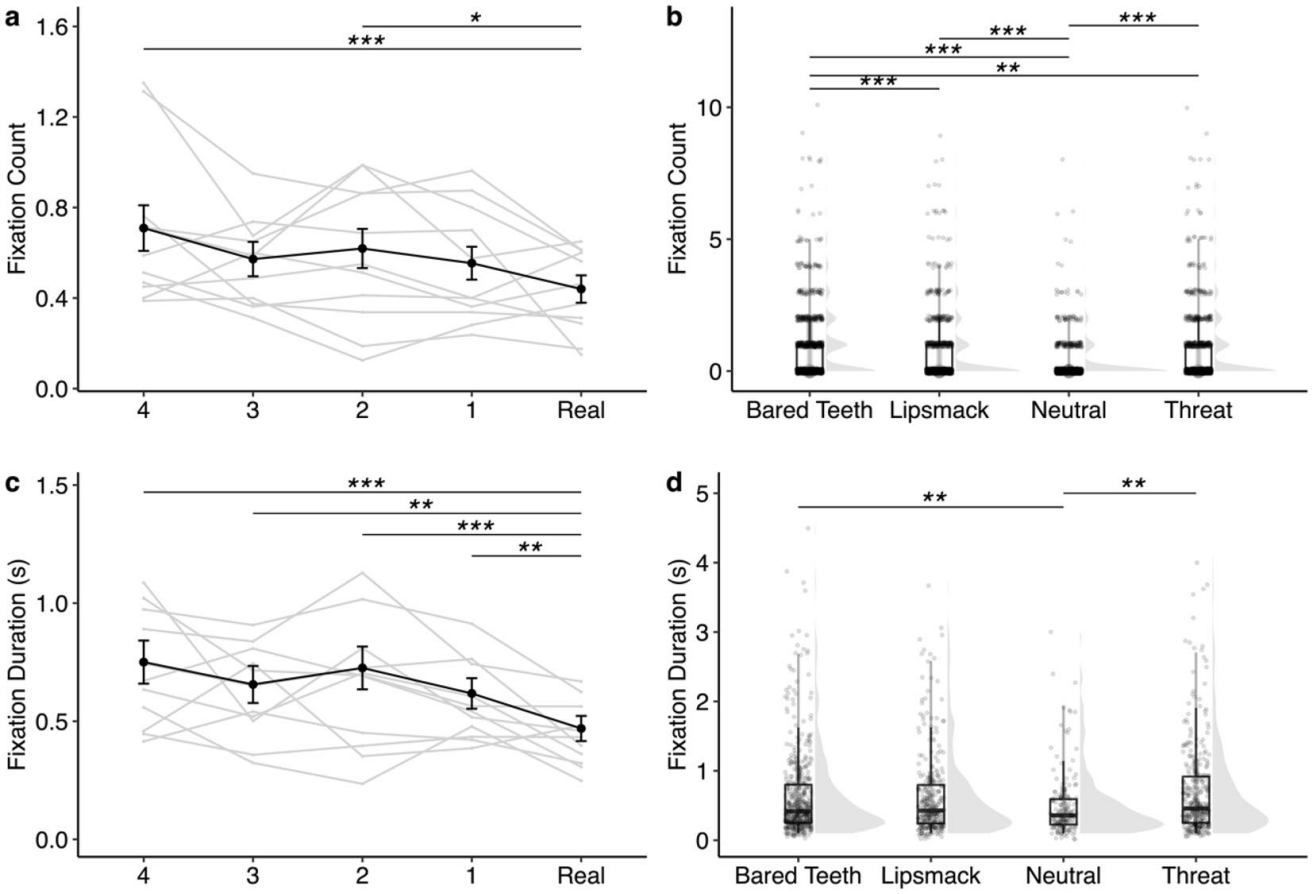
Visual attention directed toward the mouth AOI of the face stimuli. Fixation count data are in top panels and fixation duration data in bottom panels. In plots on the left (**a**,**c**), gray lines represent average behavior of a given subject pooled across facial behaviors and the black line represents average behavior across all subjects. In plots on the right (**b**,**d**), individual data points are overlaid on boxplots with violin plot density functions on the right for each facial behavior pooled across face realness levels. (**a**) Subjects looked less frequently at the mouth of Real images than Face 4 (*z* = 4.25, *p* < 0.001) or Face 2 (*z* = 3.16, *p* = 0.014). Data are summarized across 832 individual trials per face realness level. (**b**) Subjects looked at the mouth fewer times when the stimulus depicted a neutral facial behavior compared to any other facial behavior (neutral vs bared teeth: *z* = 13.77, *p* < 0.001; neutral vs lipsmack: *z* = 9.30, *p* < 0.001; neutral vs threat: *z* = − 10.46, *p* < 0.001) and more times when the facial behavior depicted was bared teeth compared to all other facial behaviors (bared teeth vs lipsmack: *z* = 6.06, *p* < 0.001; bared teeth vs threat: *z* = 3.65, *p* = 0.002). Data represent 1040 trials per facial behavior. (**c**) Subjects fixated on the mouth for less time when the image was real compared to any manipulated image (Face 1: *z* = − 3.00, *p* = 0.023; Face 2: *z* = − 4.96, *p* < 0.001; Face 3: *z* = − 3.75, *p* = 0.002; Face 4: *z* = − 5.16, *p* < 0.001). Data are averaged across 240–256 individual trials per level of face realness. (**d**) Subjects fixated on the mouth for less time when the facial behavior of the stimulus was neutral compared to either bared teeth (*z* = − 3.38, *p* = 0.004) or threat (*z* = 3.81, *p* = 0.001). Data represent 157–465 trials per facial behavior. *p < 0.05, **p < 0.01, ***p < 0.001. Error bars represent mean ± 95% confidence Interval.

Though there were several other effects of face realness on looking behavior (summarized in Table 1, Figs. 2, 3, 4, 5 and supplementary Tables S1–S12), they did not follow the pattern predicted by a UV and generally suggested that rhesus monkeys attend less to highly unrealistic eyes (Figs. 2 and 4) and more to highly unrealistic mouths (Fig. 5). When looking at a face displaying bared teeth, subjects looked at the eyes of the real image more frequently compared to the less realistic Face 3 (*z* = − 3.15, *p* = 0.014), but there were no other significant differences between stimulus realness (Fig. 2b). Similarly, subjects looked at the eyes more often in the Real image or most realistic manipulated image (Face 1) compared to the least real image Face 4 of faces depicting either a lipsmack or threat (lipsmack: Real vs Face 4: *z* = − 3.21, *p* = 0.012, Face 1 vs Face 4: *z* = − 2.92, *p* = 0.029, Fig. 2c; threat: Real vs Face 4: *z* = − 3.35, *p* = 0.007; Face 1 vs Face 4: *z* = − 2.89, *p* = 0.032; Fig. 2d), indicating reduced attention to less realistic eyes. This pattern of reduced attention toward less realistic eyes also held when looking at the duration of fixations directed toward the eyes. Subjects looked for longer at eyes in the Real image compared to either of the two least realistic faces (Faces 3 and 4) (Real vs Face 4: *z* = 4.31, *p* < 0.001; Real vs Face 3: *z* = 3.51, *p* = 0.004; Fig. 4a). Attention toward the mouth showed the opposite pattern as attention toward the eyes. Subjects fixated more times (Real vs Face 4: *z* = 4.25, *p* =  < 0.001; Real vs Face 2: *z* = 3.16, *p* = 0.014; Fig. 5a) and for longer durations (Real vs Face 4: *z* =  − 5.16, *p* < 0.001; Real vs Face 3: *z* = − 3.75, *p* = 0.002; Real vs Face 2: *z* = − 4.96, *p* < 0.001; Real vs Face 1: *z* = − 3.00, *p* = 0.023, Fig. 5c) on more manipulated mouths compared to mouths in Real images. Additionally, subjects attended to salient areas of interest depending on facial behavior as evidenced by greater attention to the mouths of face stimuli depicting facial behaviors that include exaggeration to this area (bared teeth, threat) (Fig. 5b, Fig. 5d, Table 1). Specifically, subjects looked at the mouth fewer times when the stimulus depicted a neutral facial behavior compared to any of the other facial behaviors (neutral vs bared teeth: *z* = 13.77, *p* < 0.001; neutral vs lipsmack: *z* = 9.30, *p* < 0.001; neutral vs threat: *z* = − 10.46, *p* < 0.001) and the greatest number of times when the facial behavior depicted was bared teeth compared to all other facial behaviors (bared teeth vs lipsmack: *z* = 6.06, *p* < 0.001; bared teeth vs threat: *z* = 3.65, *p* = 0.002) (Fig. 5b). Subjects also fixated on the mouth for less time when the facial behavior of the stimulus was neutral compared to either bared teeth (*z* = − 3.38, *p* = 0.004) or threat (*z* = 3.81, *p* = 0.001), both of which are facial behaviors which involve exaggeration of the mouth and which show teeth (Fig. 5d).

## Discussion

The results of our study do not demonstrate robust, if any, evidence that rhesus monkeys perceive a UV, regardless of the affect-related behaviors conveyed by the face. Theoretically, UV effects in humans occur such that the dip in affinity occurs for the very real looking stimuli^1,10^. Had we seen evidence for perception of a UV it would have manifested in monkeys directing more visual attention to the real image and more manipulated images (Faces 2 through 4) than an image that is highly realistic but has been manipulated (Face 1) thereby producing a dip, or valley, in attention toward the highly realistic stimulus, as seen in Fig. 1. It is also possible that the dip could occur elsewhere along the perceptual continuum indicating that the perception of eeriness varies in terms of its location along that continuum (e.g.,^34^) and it is reasonable to expect that species differences might drive differential locations of that dip. Attention to face stimuli was comparable across levels of face realness and in cases where there were significant differences between manipulation levels, the effects were not consistent in how subjects responded to particular manipulations (e.g., a given face realness level).

While there was not robust evidence that rhesus monkeys perceive the UV, we did find evidence that monkeys differentially direct their attention towards faces based on face realness and affect/facial behavior of the stimulus. This was true for both attention to the full-face AOI and individual areas of interest (e.g., eyes, mouth). Minimal evidence for a UV came from two places. First, subjects fixated fewer times on the eyes (and not the other regions of interest) of a neutral face (and not any of the other facial behaviors) when the most realistic manipulated image (Face 1) was shown, compared to a real face or less realistic face (Face 2), and there were no differences between the most realistic manipulated image (Face 1) and the least realistic images (Faces 3 and 4), as would be expected in a UV. The only other evidence in support of a UV effect came not from looking behavior at the faces per se but from evidence of looking behavior directed at areas of the screen that did not include the faces. Subjects did fixate on the outside portion of the stimulus more frequently for the Face 1 image compared to the real image or to the least realistic images (Face 3, Face 4). This finding may indicate that while subjects were attending to the task (the screen) they were avoiding the face stimulus when it was Face 1 manipulated, which could be interpreted as an aversion to this highly realistic manipulated image.

Interestingly, the only results suggestive of a UV effect were observed when analyzing fixation frequency, rather than fixation duration, and thus inconsistent across metrics of visual attention. Our failure to find evidence of the UV across affects and AOIs suggest that there is not a robust UV in rhesus monkeys in response to highly realistic stimuli, which stands in contrast to some previous reports of macaque behavior toward less realistic stimuli^12,28^. However, we did find that rhesus monkey behavior is impacted by face realness insofar as they do generally look more at real eyes compared to manipulated eyes and less at real mouths compared to manipulated mouths.

Looking behavior directed towards each AOI was impacted by the facial behavior depicted by the stimulus. As expected, based on previous research suggesting that rhesus monkeys alter face scanning patterns based on the behavior depicted by the face they are viewing^30,32^, we found that subjects fixated for less time on the eyes of faces depicting bared teeth displays compared to faces not depicting a behavior (neutral). This is aligned with the idea that the eyes of bared teeth displays are less important than other features—like the mouth which conveys the most salient information. Consistent with previous research^28^, low (or lesser) attention to the eyes was not observed for threat or lipsmack, whose signal value presumably also depend less on information conveyed by the eyes. Facial behavior depicted in the stimuli impacted attention to the mouth, with subjects looking fewer times at the mouth of the neutral facial behavior compared to all other facial behaviors and more times at the mouth for the bared teeth facial behavior compared to all others, a similar pattern to what has previously been observed in rhesus monkeys^28^. Despite these differences in fixation count, monkeys only fixated for less time on the mouth of the neutral face compared to threat and bared teeth. Taken together, these findings support previous research that rhesus monkeys attend to different affect displays differently^28,30,32,35^ and that attention is focused on the most salient portions of a facial stimulus which conveys the most (or most important) information for understanding the meaning of the behavior^22,30,31^, as the mouth/teeth are in a bared teeth display.

Building upon the existing reports of the presence or absence of the UV in the literature^12,27,28^, our study aimed to bring clarity to the mixed findings of a UV effect in macaque monkeys and thus variable claims about potential evolutionary origins of the UV in humans. We work with rhesus monkeys because they are an excellent model for human social behavior and psychological function in a number of domains, including social and face perception^36–40^, and thus an interesting species to evaluate possible UV effects. But they are, of course, not the only species in which we might expect to see UV-like effects. For example, other nonhuman primate species with different social structures and dynamics (e.g., even different macaque species that are more socially tolerant;^41^) may attend to social cues differently. While rhesus and long-tailed macaques are relatively similar in social organization^42^ compared to other macaque species, they do show some differences. For example, rhesus monkeys are generally more socially aggressive^43^ and are classified as being more hierarchical, nepotistic and with more asymmetry in aggression than long-tailed macaques^42^. These species-level differences in social behavior may impact sensitivity to manipulations of images of conspecifics and may underlie the differences observed in sensitivity to the UV. However, we would not expect that such subtle social differences would explain why long-tailed macaques evidenced a UV effect in one^12^, but not another^27^ study. Additionally, primates are not the only species that rely heavily on the face for social communication, leaving open the possibility that other species might also experience the UV (e.g., use of faces for social communication reviewed in:^44^; walruses and seals:^45^; dogs:^46^; horses:^47^). Understanding which species show the UV and under what conditions may bring clarity to questions about what constitutes social information and how it is extracted from stimuli and used to guide social cognition.

Considering our findings in concert with previous research on the UV in other macaques suggests some clues about how species and context differences may drive variability in UV effects. For example, the presentation of stimuli as either static or dynamic was hypothesized by Mori^1^ to alter the perception of the UV, with moving stimuli eliciting a more robust UV response than static stimuli. While we used only static images in our design, other researchers have found consistent UV effects in both long-tailed^12^ and rhesus macaques^28^ with the use of both static and dynamic stimuli. An additional variable, perhaps more important than movement, is what the stimuli actually look like in terms of realness. Some of the variability in findings regarding whether or not macaques are sensitive to the UV may be explained by differences how the face stimuli were created. For example, when higher realism (e.g., realistic coloration of body and eyes) was maintained across stimuli, there was not evidence for a UV in rhesus (our data) or long-tailed monkeys^27^. However, in studies that included unrealistic grayscale images both rhesus monkeys^28^ and long-tailed monkeys did display a UV^12^. Hairlessness alone does not explain the difference in result between those and the present study, as the stimuli used in^27^ also did not include hair but failed to induce the UV. This may suggest that to elicit a UV requires context not only in terms of a single stimulus image itself, but also in relative terms to the other information included in the stimuli set, such that experiments using only more realistic appearing faces do not elicit a UV (our data and ^27^). This variability in results suggests that while some artificial images may elicit a UV, others are unlikely to do so. In other words, small details of the stimuli used likely matter a lot.

Interestingly, the mixed UV effects across studies in monkeys (^12,27,28^, our data) are actually consistent with mixed effects observed in studies carried out with human participants. Several studies in humans have also failed to identify a UV effect^14,48,49^ (reviewed in^50^) or have found alternative patterns of behavior such as linear responses in subject-rated measures of eeriness or attractiveness, with increased human likeness generally associated with decreased eeriness^49,51^. Across these reports, there is variation in the type of stimuli used including how realistic they are and how their realism is manipulated (e.g., exaggeration of features within a single face^49^, different faces within different categories—robot vs human^34,52^) and how many levels of manipulated stimuli are presented (e.g. five all highly realistic levels^51^ vs eleven levels varying from clearly robotic all the way through real^34^). Indeed, several reports have questioned whether the UV phenomenon exists universally in humans at all or is the result of such variability in study design (e.g.,^50,53^). This discrepancy in findings across studies, both in humans and in macaque species, highlights how fragile and context dependent UV effects are and calls into question the robustness of these social perception effects across species.

Ultimately, whether or not monkeys perceive a UV has potential implications for evolutionary theory^2,3,10,12^, but also very practical implications as well. Computer-generated artificial images allow for flexible options for generating and controlling experimental stimuli including the manipulation of specific features of faces. Cues about identity^35,54^, kinship^55^ and social status^56^ are all embedded within monkey faces. Experimental manipulation of face stimuli therefore provides an avenue through which to selectively alter attributes that serve to convey important information and record behavioral and physiological responses to these manipulations. For example, by keeping face images largely structurally the same but changing the way in which a single portion of the faces (e.g., the eyes) appears, it is possible to measure the extent to which responses to facial behavior is dependent on those specific components of the face. Preparation for these kinds of experimental manipulations is already occurring in the human literature. For example, studies in humans have used facial stimuli to model individual preferences in facial attractiveness^57^, to assess the effects of facial identity (gender) on perception of emotion-related behavior^58^ and to evaluate the effect of facial structure on perception of personality traits (e.g., attractiveness, likeability, trustworthiness, competence, aggressiveness)^59,60^ and confidence in those evaluations^61^, opening the potential to use individual-level data to make manipulations specific to the preferences of a given subject. Furthermore, researchers have generated sets of artificial human faces that vary across dimensions including competence, warmth, dominance and trustworthiness^60,62^ as well as those that vary in movement cues (e.g., wrinkles) and emotional expression^63,64^, which may serve as the foundation for these kinds of studies. Similar work is being done with rhesus monkeys to generate infrastructure to use synthetic images as experimental stimuli^27,28,65^ and will serve to increase the experimental control of studies aiming to assess behavioral responses to a variety of social and facial cues. However, the utility of such manipulations is dependent on characterizing the effects of face realness on behavior. If, as we demonstrated, rhesus monkeys do not display the UV effect then the field can begin to use these techniques to address nuanced questions related to experimentally manipulated face stimuli, as the human literature is poised to do.

## Methods

### Ethics statement

All experimental procedures were noninvasive. All data presented here were collected at the California National Primate Research Center (CNPRC) under a protocol approved by the University of California, Davis, Institutional Animal Care and Use Committee, the ethics board overseeing nonhuman animal research at the university. All attempts were made to promote the psychological well-being of the animals in accordance with recommendations made by the Weatherall report, “The use of non-human primates in research.” Experimental procedures and care of the animals were consistent with the guidelines from the National Institute of Health^66^ and the recommendations in the ARRIVE guidelines.

### Subjects

Subjects were eleven male rhesus monkeys (*Macaca mulatta*), 7–11 years old (mean = 11.67, SD = 0.90), all weighing between 10–18 kg (mean = 13.79, SD = 1.87) at time of testing. Each subject was born at the CNPRC and lived for at least 2 years in a half-acre outdoor enclosure that included between 60 and 120 other monkeys. Subjects were moved indoors at an average age of 4.89 years (SD = 0.94 years). Once relocated indoors, each animal was housed in a standard adult macaque laboratory cage (66 cm width × 61 cm length × 81 cm height) and socialized with a monkey in the adjacent cage. Following standard CNPRC protocols and depending on the relationship between the two animals, daily socialization was either full, unrestrained interactions in either of the two adjoining cages or restricted to mostly visual interaction through a metal grate (moderate tactile access was also possible) for at least six hours a day. The housing room was maintained on a 12-h light/dark cycle. All animals were maintained on a diet of fresh fruit, vegetables and monkey chow (Lab Diet #5047, PMI Nutrition International Inc., Brentwood, MO), with water available ad libitum. Supplemental fruit and other foraging materials such as sunflower seeds as well as chew toys were provided daily and the room received regular enrichment in the form of other enrichment devices (tubes filled with produce) and videos.

### Face stimuli

Eighty face stimuli were created from pictures of four adult rhesus monkeys (two male, two female) from Gothard et al. (Image ID: 10, 16, 20, 70)^30^. Pictures from each identity included a neutral face (no behavior) and three facial behavioral configurations (bared teeth, threat and lipsmack). The raw, unmanipulated photographs are referred to as Real to indicate that there was zero manipulation of them. These photographs were cropped around the face and overlaid on a black background. Four different synthetic versions (Face 1, Face 2, Face 3 and Face 4) of each facial behavior for each identity were created using ZBrush (Pixologic Inc.) and Photoshop (Adobe). Stimuli are available at https://osf.io/cu8rv/. Additional information about the face stimuli can be found in Supplementary Information S1.

### Training and experimental set up

Information regarding animal training, equipment and the procedures used to carry out eye-tracking studies in our laboratory are detailed elsewhere^67–69^ and in Supplementary Information S1. Experimental stimuli were presented using a PC running the Eprime 2.0 Professional software package (Psychology Software Tools, Pittsburgh, PA). All gaze data were collected using the Eye-Trac 6.NET User Interface program (Applied Science Laboratories) on a separate PC. Infrared luminance level, pupil threshold and corneal reflection threshold were set individually for each animal at the start of each session. Sampling rate for the infrared eye-tracking camera was set to 120 Hz. A standard nine-point calibration (363 matrix of calibration stimuli) was used prior to collecting data for each animal.

### Uncanny valley experiment

On each of five test days, monkeys were presented with a different trial order with both identity and facial behavior randomized. Each session consisted of eighty trials, including all versions of each identity and facial behavior. Each trial included the same sequence of stimuli: (1) 5 s stimulus presentation, (2) 2.5 s black screen interstimulus interval (ISI), (3) a screen containing saccade stimulus (eye detection triggers continuation in trial) and (4) 2.5 s black screen ISI. As a result, the intertrial interval (ITI) time varied [ITI = 2 × 2.5 black ISI + saccade time].

### Data analysis

Applied Science Laboratories software (ASL, Applied Science Laboratories, Bedford, MA, USA) was used to collect and summarize the data. Eye tracking parameters were computed separately for multiple AOIs including the whole head, the region on the monitor but outside of the head, the mouth and the eyes. During each trial, the animal’s total number of fixations and total fixation duration were measured using the ASL Results software package. A fixation was recorded if gaze coordinates remained within 1° × 1° visual angle for at least 100 ms. The duration of a given fixation ended when gaze coordinates deviated by more than 1° × 1° visual angle for more than 360 ms. Total fixation frequency represented the cumulative number of discrete fixations that fell within the video AOI during each 5 s trial. Total fixation duration was the cumulative time (maximum = 5 s) that the animal spent fixating the video AOI.

Data analysis was carried out in R (version 4.0.2)^70^. A conceptual replication of^12^ was carried out using a Friedman’s test from the rstatix package^71^ with post hoc analyses conducted with a Dunn’s post hoc test with corrections for multiple comparisons from the dunn.test package^72^. Generalized linear mixed effects models implemented in the *lme4* package were used to model number of fixations and fixation durations, accounting for within-subject clustering of observations by incorporating a random intercept for each subject. The *DHARMa* package^73^ was used to generate Q-Q plots for the fitted model residuals against simulated residuals to evaluate model fit for selecting appropriate glms (e.g., negative binomial vs Poisson). Poc hoc testing was done using the Tukey method of adjustment for multiple comparisons. All statistical analyses were two tailed and used an alpha level of 0.05. Figures were produced using the esquisse^74^ and ggplot2 packages^75^.

## Supplementary Information

Supplementary Information.

## Data Availability

The datasets generated during and/or analyzed during the current study are available from the corresponding author on reasonable request.
