# Supplementary material for: Monkey visual attention does not fall into the uncanny valley
Source: Sci Rep. 2022 Jul 11;12:11760. doi: 10.1038/s41598-022-14615-x (PMC9273626; doi:10.1038/s41598-022-14615-x)
Supplement: Supplementary file 1 — Supplementary Information. [file 41598_2022_14615_MOESM1_ESM.pdf]

Supplementary Information

Monkey visual attention does not fall into the uncanny valley

Sarah B. Carp*[1], Anthony C. Santistevan[1,2], Christopher J. Machado[1,3], Alexander M. Whitaker[1], Brittany L. Aguilar[1], Eliza Bliss-Moreau*[1,2]

[1]California National Primate Research Center, Davis, CA 95616
[2]Department of Psychology, University of California Davis, Davis CA 95616
[3]Department of Psychiatry and Behavioral Sciences, University of California Davis, Davis CA 95616

Methods

Face Stimuli: Creating the computer-generated synthetic images proceeded via a series of steps. First, a digital skull model was created to serve as a template for the synthetic heads. To do this, a rhesus monkey was photographed from different angles (head-on, from above, and in profile), these images were imported into ZBrush, and compiled into a 3D model within the application. Depending on the quality of the 3D scan, the retopology tool ZRemesher was used to create a symmetrical model and parts of the skull were sculpted, including the teeth. The skin-painting tool was then used to fill in space around the skull, creating the flesh of the head. A photo of a rhesus monkey with its whole head shaved was used a reference for skin elaboration. Following addition of skin, the eyes were colored, and hairs were formed around the head.

To create the Face 1 version of the stimuli, unmanipulated real image masks were overlaid on top of the head template, hair and feature details were individualized, and the Best Possible Render (BPR) setting was applied. To create the Face 2 version of the stimuli, the Best Possible Render (BPR) setting was eliminated from the Face 1 settings. To create Face 3 versions of the stimuli, the mask was removed from Face 2 and replaced with a mesh texture (SketchShaded), and the color was standardized (Red :173, Green:139, Blue:112). To create the Face 4 versions of the stimuli, the Face 3 model was reduced in polygon count to ¼ the original count. All images were exported to be isolated and placed on a black background.

Training and Experimental Setup: All training and testing occurred while the animals sat in a modified primate chair with a slanted top (Crist Instrument Co., Inc., Damascus, MD). Each animal was fit with a customized thermoplastic face mask (Civco Medical Solutions, Kalona, IA; 24.8 cm wide × 28 cm long × 3.2 mm thick) that was secured to the top of the chair, allowing for precise eye tracking [1,2]. Each animal was habituated to sitting in the primate chair and wearing his mask for successively longer periods until they were comfortable for up to 90 minutes. After habituation to the face mask, each chaired animal was rolled into a sound-attenuating testing chamber (Acoustic Systems, Austin, TX; 2.1 m wide x 2.4 m tall x 1.1 m deep) for habituation to this testing context and the video eye-tracker (Applied Science Laboratories, Bedford, MA; model R-HS-S6). A wide-screen, color video monitor (60.96 cm diagonal; Gateway Inc., Irvine, CA; model LP2424) was positioned at the monkey's eye level. The video monitor was positioned 127 cm from the animals' eyes, while the eye-tracking camera was positioned on a tripod 53.34 cm from the animals' eyes. A curved mouthpiece (Crist Instrument Co., Inc.; model # 5-RLD-00A) was attached to the top-left of the chair and connected to an automatic juice dispenser (Crist Instrument Co., Inc.; model # 5-RLD-E3) so that juice reward could be dispensed

throughout the testing session. A white noise generator (60 dB) inside of the chamber was used to mask outside auditory distractions.

Calibration stimuli were videos presented in small portions of the screen (8.89 × 5.72 cm on screen, 4° visual angle) of rhesus monkeys from the outdoor housing enclosures at the CNPRC. This allows for each animal's gaze to be attracted to different portions of the screen in order to calibrate the data acquisition software.

Once reliably calibrated, each monkey was trained to fixate color animated GIF images at random spatial positions on the computer screen for juice rewards that were manually dispensed by the experimenter. Animals completed this phase of training once they fixated on the GIF images consistently for 2 consecutive days. During the second phase of fixation training, animals viewed either photographs (color or black and white; 5-second duration) on a 50% gray background or color video clips (taken from commercial movies and nature documentary DVDs; 30-second duration) on a black background. Each photograph or movie was separated by four 50% gray screens: 1) blank, 10-second duration, 2) black square target (3.4º visual angle) at center, 3) same black square target positioned randomly at 1 of 8 points around the screen periphery, and 4) blank, 10-second duration. Animals were required to fixate each black square target for at least 250 ms to obtain a small juice reward and move on to the next picture or movie trial, thus ensuring accuracy of the point-of-gaze data throughout a prolonged testing session. The animals completed the final phase of training once they finished 100 picture or 50 movie trials in less than 90 minutes on 3 consecutive days. Once trained and prior to data collection for the current study, animals completed a longer experimental task in which they watched 600 30-second videos of other monkeys and natural scenes[3–5].

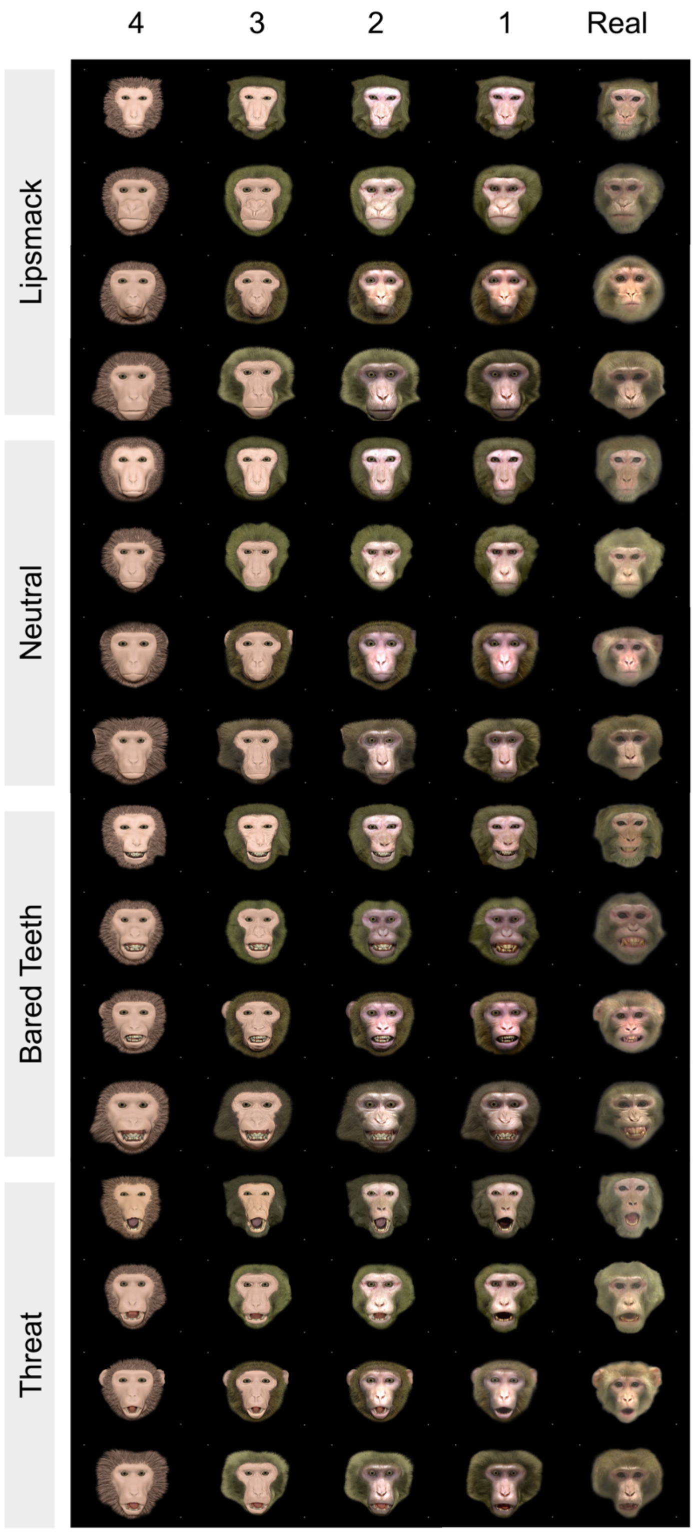

Figure S1. The 80 face stimuli used in this experiment. Images of four individual monkeys displaying four distinct facial behaviors (lipsmack, neutral, bared teeth, threat) were manipulated to create five levels of varying realness from least realistic (4) to most realistic artificial (1) and finally to the unmanipulated Real image.

Table S1. Main Effects for Eye AOI with Frequency of Fixations as the Outcome Variable

	χ^2	**df**	**p**
Face Realness	40.830	4	<0.001
Affect	32.297	3	<0.001
Day	166.733	4	<0.001
Face Realness x Facial Behavior	30.360	12	0.002

Type II Wald Chi-square test

Table S2. Post Hocs for Eye AOI with Frequency of Fixations as the Outcome Variable

Comparison of Test Day				
**Comparison**	**Ratio**	**SE**	**z**	**p**
Day 1/2	1.458	0.0716	7.685	<0.001
Day 1/3	1.689	0.089	10.001	<0.001
Day 1/4	1.564	0.081	8.608	<0.001
Day 1/5	1.813	0.097	11.167	<0.001
Day 2/3	1.158	0.063	2.721	0.051
Day 2/4	1.072	0.057	1.303	0.689
Day 2/5	1.243	0.068	3.960	<0.001
Day 3/4	0.926	0.052	-1.386	0.637
Day 3/5	1.073	0.061	1.241	0.728
Day 4/5	1.159	0.066	2.614	0.068
**Face Realness x Facial Behavior Post Hoc Tests – Bared Teeth**				
**Comparison**	**Ratio**	**SE**	**z**	**p**
Face 4 vs Face 3	1.313	0.149	2.404	0.111
Face 4 vs Face 2	1.071	0.117	0.631	0.970
Face 4 vs Face 1	0.980	0.106	-0.186	1.000
Face 4 vs Real	0.923	0.098	-0.754	0.944
Face 3 vs Face 2	0.816	0.093	-1.782	0.384
Face 3 vs Face 1	0.746	0.085	-2.580	0.074
Face 3 vs Real	0.703	0.079	-3.149	0.014
Face 2 vs Face 1	0.915	0.100	-0.815	0.926
Face 2 vs Real	0.861	0.093	-1.385	0.637
Face 1 vs Real	0.942	0.101	-0.564	0.980
**Face Realness x Facial Behavior Post Hoc Tests – Lipsmack**				
**Comparison**	**Ratio**	**SE**	**z**	**p**
Face 4 vs Face 3	0.839	0.090	-1.634	0.476
Face 4 vs Face 2	0.919	0.100	-0.777	0.937
Face 4 vs Face 1	0.735	0.077	-2.923	0.029
Face 4 vs Real	0.714	0.075	-3.207	0.012

Face 3 vs Face 2	1.095	0.116	0.856	0.913
Face 3 vs Face 1	0.876	0.090	-1.293	0.696
Face 3 vs Real	0.852	0.087	-1.578	0.512
Face 2 vs Face 1	0.800	0.083	-2.145	0.201
Face 2 vs Real	0.778	0.081	-2.429	0.108
Face 1 vs Real	0.972	0.097	-0.284	0.999

Face Realness x Facial Behavior Post Hoc Tests – Neutral

Comparison	Ratio	SE	z	p
Face 4 vs Face 3	0.795	0.083	-2.195	0.182
Face 4 vs Face 2	0.727	0.076	-3.062	0.019
Face 4 vs Face 1	0.966	0.104	-0.322	0.998
Face 4 vs Real	0.638	0.065	-4.407	<0.001
Face 3 vs Face 2	0.915	0.092	-0.887	0.902
Face 3 vs Face 1	1.215	0.126	1.879	0.329
Face 3 vs Real	0.802	0.079	-2.247	0.163
Face 2 vs Face 1	1.328	0.137	2.751	0.047
Face 2 vs Real	0.877	0.085	-1.351	0.659
Face 1 vs Real	0.660	0.067	-4.102	<0.001

Face Realness x Facial Behavior Post Hoc Tests – Threat

Comparison	Ratio	SE	z	p
Face 4 vs Face 3	0.946	0.106	-0.493	0.988
Face 4 vs Face 2	0.835	0.092	-1.634	0.476
Face 4 vs Face 1	0.731	0.079	-2.886	0.032
Face 4 vs Real	0.698	0.075	-3.351	0.007
Face 3 vs Face 2	0.883	0.097	-1.141	0.785
Face 3 vs Face 1	0.773	0.083	-2.397	0.116
Face 3 vs Real	0.737	0.079	-2.862	0.034
Face 2 vs Face 1	0.875	0.092	-1.261	0.715
Face 2 vs Real	0.835	0.087	-1.723	0.419
Face 1 vs Real	0.954	0.098	-0.458	0.991

p-value adjustment: Tukey method

Table S3. Main Effects for Eye AOI with Fixation Duration as the Outcome Variable

	χ^2	df	p
Face Realness	23.818	4	<0.001
Affect	13.090	3	0.004
Day	52.605	4	<0.001
Face Realness x Facial Behavior	18.697	12	0.096

Type II Wald Chi-square test

Table S4. Post Hocs for Eye AOI with Fixation Duration as the Outcome Variable

Comparison of Test Day				
**Comparison**	**Estimate**	**SE**	**z**	**p**
Day 1/2	-0.174	0.063	-2.758	0.046
Day 1/3	-0.356	0.072	-4.959	<0.001
Day 1/4	-0.398	0.072	-5.511	<0.001
Day 1/5	-0.420	0.078	-5.403	<0.001
Day 2/3	-0.182	0.077	-2.363	0.126
Day 2/4	-0.224	0.077	-2.899	0.031
Day 2/5	-0.246	0.083	-2.976	0.024
Day 3/4	-0.042	0.084	-0.507	0.987
Day 3/5	-0.065	0.089	-0.728	0.950
Day 4/5	-0.022	0.089	-0.249	0.999
**Comparison of Face Realness**				
**Comparison**	**Estimate**	**SE**	**z**	**p**
Face 4 vs Face 3	0.068	0.084	0.803	0.930
Face 4 vs Face 2	0.176	0.081	2.181	0.187
Face 4 vs Face 1	0.267	0.078	3.406	0.006
Face 4 vs Real	0.327	0.076	4.314	<0.001
Face 3 vs Face 2	0.109	0.079	1.376	0.643
Face 3 vs Face 1	0.199	0.076	2.605	0.070
Face 3 vs Real	0.259	0.074	3.513	0.004
Face 2 vs Face 1	0.090	0.073	1.241	0.727
Face 2 vs Real	0.151	0.070	2.149	0.199
Face 1 vs Real	0.060	0.067	0.899	0.897
**Comparison of Facial Behavior**				
**Comparison**	**Estimate**	**SE**	**z**	**p**
Bared teeth vs lipsmack	0.178	0.070	2.528	0.056
Bared teeth vs neutral	0.232	0.068	3.399	0.004
Bared teeth vs threat	0.066	0.074	0.900	0.805
Lipsmack vs neutral	0.054	0.062	0.873	0.819

Lipsmack vs threat	-0.112	0.068	-1.648	0.352
Neutral vs threat	-0.166	0.066	-2.528	0.056

p-value adjustment: Tukey method

Table S5. Main Effects for Mouth AOI with Frequency of Fixations as the Outcome Variable

	χ^2	**df**	**p**
Face Realness	24.250	4	<0.001
Affect	197.239	3	<0.001
Day	26.305	4	<0.001
Face Realness x Facial Behavior	5.746	12	0.928

Type II Wald Chi-square test

Table S6. Post Hocs for Mouth AOI with Frequency of Fixations as the Outcome Variable

Comparison of Test Day				
**Comparison**	**Ratio**	**SE**	**z**	**p**
Day 1/2	0.787	0.071	-2.649	0.062
Day 1/3	1.206	0.118	1.916	0.308
Day 1/4	1.153	0.112	1.466	0.585
Day 1/5	1.108	0.108	1.047	0.833
Day 2/3	1.533	0.147	4.450	<0.001
Day 2/4	1.466	0.140	4.006	<0.001
Day 2/5	1.408	0.135	3.569	<0.001
Day 3/4	0.956	0.097	-0.445	0.992
Day 3/5	0.918	0.093	-0.842	0.918
Day 4/5	0.961	0.097	-0.400	0.995
**Comparison of Face Realness**				
**Comparison**	**Ratio**	**SE**	**z**	**p**
Face 4 vs Face 3	1.180	0.115	1.695	0.437
Face 4 vs Face 2	1.114	0.108	1.119	0.797
Face 4 vs Face 1	1.270	0.125	2.427	0.108
Face 4 vs Real	1.535	0.154	4.248	<0.001
Face 3 vs Face 2	0.944	0.093	-0.588	0.977
Face 3 vs Face 1	1.076	0.108	0.733	0.949
Face 3 vs Real	1.300	0.133	2.562	0.078
Face 2 vs Face 1	1.140	0.113	1.326	0.675
Face 2 vs Real	1.378	0.140	3.162	0.014
Face 1 vs Real	1.208	0.125	1.834	0.354
**Comparison of Facial Behavior**				
**Comparison**	**Ratio**	**SE**	**z**	**p**
Bared teeth vs lipsmack	1.634	0.133	6.057	<0.001
Bared teeth vs neutral	3.684	0.349	13.773	<0.001
Bared teeth vs threat	1.336	0.106	3.647	0.002
Lipsmack vs neutral	2.254	0.221	8.295	<0.001

Lipsmack vs threat	0.817	0.069	-2.405	0.076
Neutral vs threat	0.363	0.035	-10.460	<0.001

p-value adjustment: Tukey method

Table S7. Main Effects for Mouth AOI with Fixation Duration as Outcome Variable

	**χ^2**	**df**	**p**
Face Realness	47.542	4	<0.001
Affect	17.081	3	<0.001
Day	7.744	4	0.101
Face Realness x Facial Behavior	6.950	12	0.861

Type II Wald Chi-square test

Table S8. Post Hocs for Mouth AOI with Fixation Duration as the Outcome Variable

Comparison of Test Day				
**Comparison**	**Estimate**	**SE**	**z**	**p**
Day 1/2	0.082	0.017	4.887	<0.001
Day 1/3	0.108	0.017	6.554	<0.001
Day 1/4	0.122	0.016	7.502	<0.001
Day 1/5	0.098	0.017	5.912	<0.001
Day 2/3	0.027	0.015	1.775	0.388
Day 2/4	0.041	0.015	2.756	0.046
Day 2/5	0.017	0.015	1.097	0.808
Day 3/4	0.014	0.014	0.979	0.865
Day 3/5	-0.010	0.015	-0.685	0.960
Day 4/5	-0.024	0.014	-1.668	0.454
**Comparison of Face Realness**				
**Comparison**	**Estimate**	**SE**	**z**	**p**
Face 4 vs Face 3	-0.183	0.120	-1.518	0.551
Face 4 vs Face 2	-0.054	0.110	-0.491	0.988
Face 4 vs Face 1	-0.285	0.121	-2.354	0.128
Face 4 vs Real	-0.708	0.137	-5.156	<0.001
Face 3 vs Face 2	0.128	0.114	1.124	0.794
Face 3 vs Face 1	-0.102	0.124	-0.821	0.924
Face 3 vs Real	-0.525	0.140	-3.746	0.002
Face 2 vs Face 1	-0.231	0.115	-2.010	0.261
Face 2 vs Real	-0.653	0.132	-4.958	<0.001
Face 1 vs Real	-0.423	0.141	-3.001	0.023
**Comparison of Facial Behavior**				
**Comparison**	**Estimate**	**SE**	**z**	**p**
Bared teeth vs lipsmack	-0.087	0.089	-0.972	0.766
Bared teeth vs neutral	-0.436	0.129	-3.377	0.004
Bared teeth vs threat	0.069	0.082	0.841	0.835
Lipsmack vs neutral	-0.349	0.137	-2.554	0.052

Lipsmack vs threat	0.155	0.094	1.654	0.349
Neutral vs threat	0.504	0.132	3.813	<0.001

p-value adjustment: Tukey method

Table S9. Main Effects for Screen but not Face (“Outside”) AOI with Frequency of Fixations as the Outcome Variable

	χ^2	**df**	**p**
Face Realness	23.165	4	<0.001
Affect	9.697	3	0.021
Day	128.340	4	<0.001
Face Realness x Facial Behavior	19.375	12	0.080

Type II Wald Chi-square test

Table S10. Post Hocs for Screen but not Face ("Outside") AOI with Frequency of Fixations as the Outcome Variable

Comparison of Test Day				
**Comparison**	**Ratio**	**SE**	**z**	**p**
Day 1/2	0.860	0.024	-5.332	<0.001
Day 1/3	0.886	0.026	-4.145	<0.001
Day 1/4	0.771	0.022	-9.122	<0.001
Day 1/5	0.753	0.022	-9.942	<0.001
Day 2/3	1.030	0.030	1.044	0.835
Day 2/4	0.896	0.025	-3.919	0.001
Day 2/5	0.875	0.025	-4.748	<0.001
Day 3/4	0.869	0.025	-4.922	<0.001
Day 3/5	0.849	0.024	-5.742	<0.001
Day 4/5	0.977	0.027	-0.826	0.923
**Comparison of Face Realness**				
**Comparison**	**Ratio**	**SE**	**z**	**p**
Face 4 vs Face 3	0.957	0.027	-1.537	0.583
Face 4 vs Face 2	0.917	0.026	-3.055	0.019
Face 4 vs Face 1	0.885	0.025	-4.337	<0.001
Face 4 vs Real	0.969	0.028	-1.114	0.799
Face 3 vs Face 2	0.958	0.027	-1.519	0.550
Face 3 vs Face 1	0.924	0.026	-2.803	0.041
Face 3 vs Real	1.012	0.029	0.424	0.993
Face 2 vs Face 1	0.965	0.027	-1.286	0.700
Face 2 vs Real	1.056	0.030	1.943	0.294
Face 1 vs Real	1.095	0.031	3.229	0.011
**Comparison of Facial Behavior**				
**Comparison**	**Ratio**	**SE**	**z**	**p**
Bared teeth vs lipsmack	0.979	0.025	-0.843	0.834
Bared teeth vs neutral	0.929	0.024	-2.909	0.019
Bared teeth vs threat	0.954	0.024	-1.852	0.249

Lipsmack vs neutral	0.949	0.024	-2.069	0.163
Lipsmack vs threat	0.975	0.025	-1.010	0.744
Neutral vs threat	1.027	0.026	1.059	0.715

p-value adjustment: Tukey method

Table S11. Main Effects for Screen but not Face ("Outside") AOI with Fixation Duration as the Outcome Variable

	χ^2	**df**	**p**
Face Realness	4.005	4	0.405
Affect	8.503	3	0.037
Day	63.038	4	<0.001
Face Realness x Facial Behavior	13.027	12	0.367

Type II Wald Chi-square test

Table S12. Post Hocs for Screen but not Face ("Outside") AOI with Fixation Duration as the Outcome Variable

Comparison of Test Day				
**Comparison**	**Estimate**	**SE**	**z**	**p**
Day 1/2	0.082	0.017	4.887	<0.001
Day 1/3	0.108	0.017	6.554	<0.001
Day 1/4	0.122	0.016	7.502	<0.001
Day 1/5	0.098	0.017	5.912	<0.001
Day 2/3	0.027	0.015	1.775	0.388
Day 2/4	0.041	0.015	2.756	0.046
Day 2/5	0.017	0.015	1.097	0.808
Day 3/4	0.014	0.014	0.979	0.865
Day 3/5	-0.010	0.015	-0.685	0.960
Day 4/5	-0.024	0.014	-1.668	0.454
**Comparison of Facial Behavior**				
**Comparison**	**Estimate**	**SE**	**z**	**p**
Bared teeth vs lipsmack	0.015	0.014	1.082	0.701
Bared teeth vs neutral	0.037	0.014	2.697	0.035
Bared teeth vs threat	0.028	0.014	2.040	0.173
Lipsmack vs neutral	0.022	0.013	1.616	0.369
Lipsmack vs threat	0.013	0.014	0.957	0.774
Neutral vs threat	-0.009	0.013	-0.662	0.911

p-value adjustment: Tukey method

Supplementary Information References

1. Bliss-Moreau, E., Theil, J. H. & Moadab, G. Efficient cooperative restraint training with rhesus macaques. *Journal of Applied Animal Welfare Science* **16**, 98–117 (2013).
2. Machado, C. J. & Nelson, E. E. Eye-tracking with nonhuman primates is now more accessible than ever before. *Am. J. Primatol.* **73**, 562–569 (2011).
3. Machado, C. J., Bliss-Moreau, E., Platt, M. L. & Amaral, D. G. Social and nonsocial content differentially modulates visual attention and autonomic arousal in rhesus macaques. *PLoS ONE* **6**, 10 (2011).
4. Bliss-Moreau, E., Machado, C. J. & Amaral, D. G. Macaque cardiac physiology is sensitive to the valence of passively viewed sensory stimuli. *PLoS ONE* **8**, e71170 (2013).
5. Bliss-Moreau, E., Moadab, G. & Machado, C. J. Monkeys preferentially process body information while viewing affective displays. *Emotion* **17**, 765–771 (2017).
